# Traumatic Glaucoma in Children

**DOI:** 10.5005/jp-journals-10008-1162

**Published:** 2014-06-12

**Authors:** Savleen Kaur, Sushmita Kaushik, Surinder Singh Pandav

**Affiliations:** Senior Resident, Advanced Eye Centre, Postgraduate Institute of Medical Education and Research, Chandigarh, India; Assocaite Professor, Advanced Eye Centre, Postgraduate Institute of Medical Education and Research, Chandigarh, India; Professor, Advanced Eye Centre, Postgraduate Institute of Medical Education and Research, Chandigarh, India

**Keywords:** Trauma, Childhood, Glaucoma, Penetrating, Blunt.

## Abstract

Young patients are more prone to ocular trauma but most of the published studies describe complicated cataract as a result of trauma with its treatment modality. As a result, little is known about the different causes, common presenting signs and symptoms, visual outcomes, and most frequent management modalities of traumatic glaucoma in children. This review aims to study the demographical profile, presentation, management and outcome of traumatic glaucoma in children as well as the various factors associated with advanced glaucomatous changes.

**How to cite this article:** Kaur S, Kaushik S, Pandav SS. Traumatic Glaucoma in Children. J Curr Glaucoma Pract 2014; 8(2):58-62.

## INTRODUCTION

Ocular injuries comprise a large part of avoidable blindness. This loss is more in children owing to injuries sustained during play. This age group is more susceptible to losing vision due to the compounding factor of amblyopia especially if appropriate management is not constituted in time. 160,000 to 280,000 of children less than 15 years of age every year sustain ocular trauma serious enough to require hospi ta-lization.^1,2^

A total of 95% of ocular injuries do not require admission. Injuries not requiring hospitalization but causing serious ocular morbidity to children under age 15 years is 3.3-5.7 million annually.^3,4^ Such a large burden of trauma both penetrating and nonpenetrating leads to various sight threatening complications. There is paucity of literature on the worldwide incidence of glaucoma in the pediatric age group due to trauma. The profile and management of traumatic glaucomas has been well-described in adults.^2,5^ Young patients are more prone to ocular trauma but most of the published studies describe complicated cataract as a result of trauma with its treatment modality. As a result, little is known about the different causes, common presenting signs and symptoms, visual outcomes, and most frequent management modalities of traumatic glaucoma in children. This chapter aims to study the demographical profile, presentation, management and outcome of traumatic glaucoma in children as well as the various factors associated with advanced glaucomatous changes.

### Definition of Traumatic Glaucoma

The definition is quite vague, usually defined by the judgment of the treating physician. Any post-trauma raised intraocular pressure (IOP) more than 21 mm Hg post-trauma which may be acute or chronic in onset, and blunt or penetrating in nature, in children less than 12 years of age supplemented with/without establishment of glaucomatous optic neuropathy on visual field testing wherever possible is the acceptable definition.

### Incidence

Data from the United States eye injury registry (USEIR) was obtained from 3,627 patients who experienced penetrating ocular trauma in a large scale study,^6^ found an overall incidence of post-traumatic glaucoma after penetrating ocular involvement of 2.67%. However, they did not describe the incidence in the pediatric age group per se.

### Age

Most of the studies report the age of various injuries in children depending on the type of activity involved. Very small children less than 2 years of age do not incur injuries related to outdoor activities or injuries which are sports related. The incidence remains stable from age 2 to 12 years.^7^ Toy injuries are commonest in 2 to 4 years and injury from falls commonest in those learning to walk (less than 5 years).

### Gender distribution

All studies show traumatic injuries to be more in male children. Hence, the incidence of traumatic glaucoma.^3,8^

### Type of Injury

Sports account for about 27% of all pediatric ocular trauma requiring hospital admission in the United States.^9^ In northwest India, 45% of sport-related injuries were from Gilli-Danda or bows and arrows.^10^

### Clinical Features

Because of the inability of children to verbalize complaints and because of the immaturity of the visual system, children may be more likely to manifest strabismus or nystagmus in the setting of blunt trauma. In children, the most common finding following ocular trauma is hyphema, and it occurs in 32% of all traumas.^11,12^ Authors have reported the incidence of traumatic hyphema seen in children or young adults of approximately two per 10,000 children per year.^13^

## MECHANISM

### Blunt Trauma

**Fig. 1:**
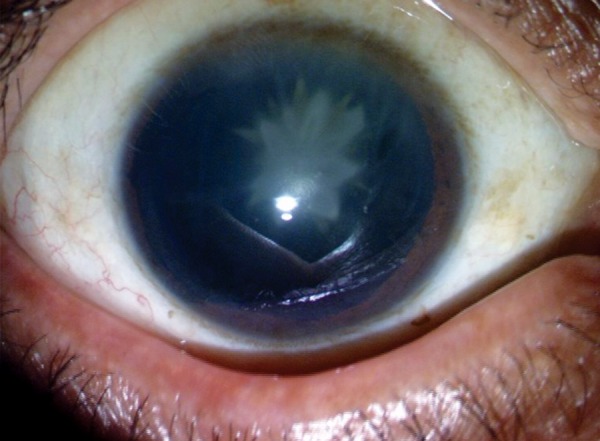
Blunt ocular trauma in a child causing a rosette cataract and traumatic glaucoma

Mechanism of raised IOP in blunt trauma (Fig. 1) includes the following:

Uveitis

The initial IOP is often low due to traumatic iritis causing a transient ciliary body shutdown. The IOP elevation occurring after a few days is usually mild and easily controlled. The mechanism is secondary to obstruction of trabecular meshwork by Inflammatory debris as well as swelling of the trabecular meshwork contributing to the outfow obstruction.

Hyphema

**Fig. 2:**
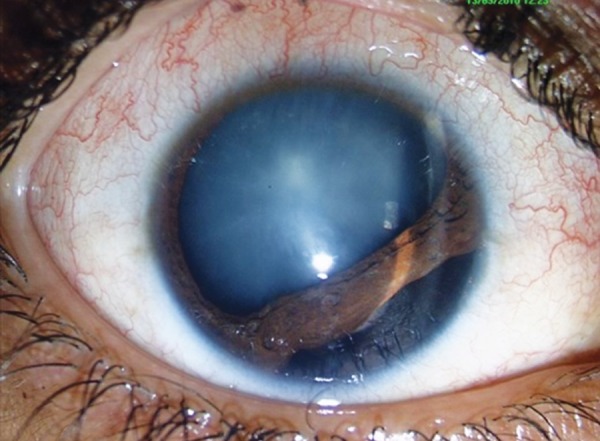
Blunt ocular trauma in child causing cataract and iridodialysis

Blunt injury results in anteroposterior compression of the globe and equatorial globe expansion which induces stress in the anterior chamber angle structures. This in turn leads to iris (Fig. 2) and ciliary body vessel rupture with subsequent hemorrhage.

Acutely, the intraocular pressure is raised due to:

1. Occlusion of the trabecular meshwork by clot, infa m-matory cells, or red blood cells
2. Pupillary block secondary to a clot involving both the anterior and posterior chambers.

Usually the larger the hyphema the greater the chance of raised IOP but in patients with sickle cell disease high IOP can be seen with a small hyphema. Usually visual prognosis and complications are worse in the presence of total hyphema as opposed to subtotal hyphema.^1^ Eyes with total hyphemas are more likely to require surgery than the eyes with partial hyphemas.

### Rebleed in Hyphema

**Fig. 3:**
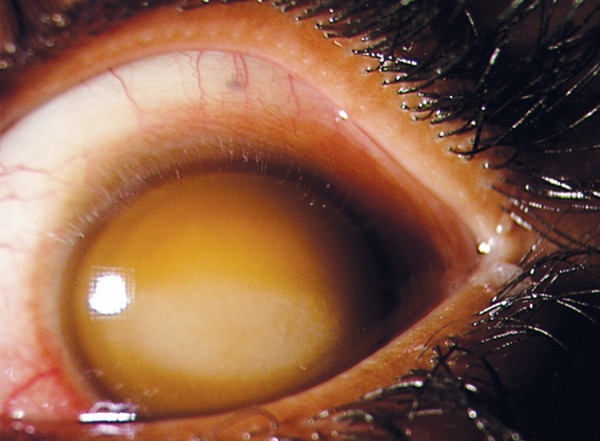
Corneal blood staining after traumatic total hyphema

Rebleeding further due to clot lysis or retraction is associated with raised IOP. Since a rebleed can cause a substantial increase in hyphema size, rebleeding may be associated with complications such as increased intraocular pressure, corneal bloodstaining (Fig. 3), optic atrophy, and peripheral anterior synechiae.

The incidence of surgical intervention is higher in rebleeds. The risk of rebleed is, however, not related to the size of hyphema.

### Ghost Cell Glaucoma

After vitreous hemorrhage, erythrocytes degenerate from biconvex cells into spherical khaki colored ghost cells and if the anterior hyaloid face is ruptured, these ghost cells can migrate into the anterior chamber and obstruct the trabecular meshwork. Red blood cells take 1 to 2 weeks to degenerate into ghost cells so the onset of ghost cell glaucoma is usually around 3 weeks post-trauma. It is rare in children and in one review of over 200 cases of traumatic and nontraumatic vitreous hemorrhage in children, no case of ghost cell glaucoma was reported.^14^

### Angle Recession

**Fig. 4:**
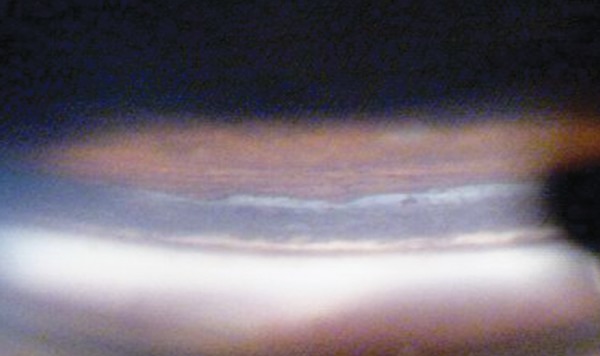
Angle recession of the superior angle viewed in the inferior mirror

The incidence of angle recession (Fig. 4) after eye trauma ranges from 20 to 94%.

A total of 5 to 20% of patients with traumatic angle recession will go on to develop glaucoma. The possibility of developing glaucoma in an eye with angle recession appears to be related to the extent of angle recession. If more than 180 of the anterior chamber angle is involved, there is a greater chance of subsequently developing glaucoma.

### Dislocated Lens

Total anterior dislocation of lens can lead to angle closure glaucoma secondary to pupillary block. Posterior dislocation is less likely to cause glaucoma but if the vitreous prolapses, pupillary block glaucoma may ensue or if the dislocated lens becomes cataractous and lens proteins leak, phacolytic glaucoma may also occur.

### Vitreous Hemorrhage

Traumatic causes account for 73.1% of all vitreous hemorrhage in children in a large scale study.^14^ These included non penetrating trauma (29.6%), penetrating trauma (24.7%), shaken baby syndrome (8.6%), postocular surgery (5.4%), and birth trauma (4.8%).

## PENETRATING TRAUMA

**Fig. 5:**
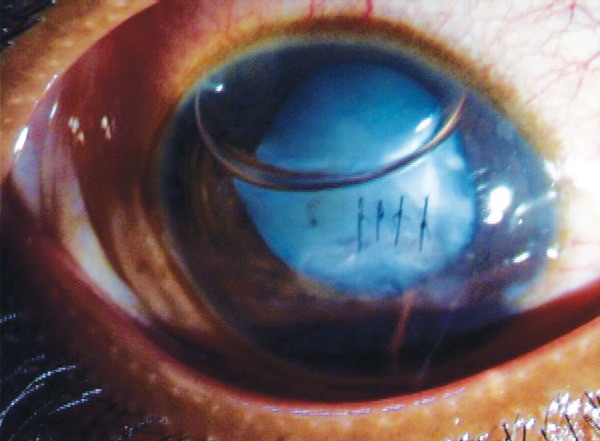
Repaired traumatic corneal laceration

Initial IOP is usually low after penetrating or perforating injury, but after wound closure, glaucoma may develop (Fig. 5). The causes include:

1. Structural alterations.
2. Blockage of the trabecular meshwork by blood cells from a hyphema, Inflammatory debris, lens particles, or ghost cells.
3. Secondary responses such as Inflammation- may lead to synechial closure of the angle.
4. Angle closure may occur due to iris bombe synechial closure at the pupillary margin, or lens swelling.
5. Long-standing vitreous hemorrhage.

## MANAGEMENT OF RAISED IOP AFTER TRAUMA

- Hyphema patients need to be followed daily for 3 to 5 days to monitor for IOP and rebleed.
- IOP over 21 should be treated.
- Even after stabilization of IOP after the acute phase is over, these children might need a lifelong follow-up to look for angle recession.

### Topical

Unless there are contraindications, beta blockers are usually used first. Adrenergic agonists such as apraclonidine or brimonidine are avoided in young children. Topical CAI's can also be added if needed, even in patients with sickle cell disease. Pilocarpine is avoided as this increases vascular permeability and excarbates pupillary block. Prostaglandin analogues cause an increased Inflammatory response in such circumstances and should be avoided. The use of cyclo plegics reduces the ciliary spasm and also reduces the incidence of occlusio pupillae. However, longer acting cycloplegics are usually indicated in the setting of hyphema to reduce the pupillary rubbing and decrease the chances of rebleed.

### Systemic

In cases of sickle cell disease the use of systemic acetazola-mide can cause a sickle crisis. Its use in sickle cell trait is not contraindicated but the child should be well hydrated. Intra venous mannitol needs hematological surveillance in the presence of sickle cell disease.

### Surgical

In cases of total hyphema or partial hyphema with sustained IOP greater than 30 mm Hg for 3 days on maximum medical therapy or any evidence of corneal staining, surgical washout of the anterior chamber is usually performed.

Any condition causing a persistent raised IOP which is medically uncontrolled calls for a trabeculectomy.

### Risk Factors

The following are the risk factors for a greater chance of developing glaucoma after ocular trauma reported in various clinical studies:

1. Advancing age
2. Lens injury
3. Baseline visual acuity below 20/200
4. Anterior chamber Inflammation
5. Zone II injury
6. Penetrating ocular injury,
7. Presence of vitreous hemorrhage
8. Presence of an intraocular foreign body were significant risk factors for developing post-traumatic glaucoma in a recent study in the African subcontinenet.^5^
9. Sihota et al^15^ reported increased pigmentation of the angle on gonioscopic findings, a higher baseline IOP, the absence of a cyclodialysis cleft on UBM or gonioscopic findings, hyphema, angle recession >180° and lens injury to be predictors of post-traumatic glaucoma.

## TRAUMATIC GLAUCOMA AT A TERTIARY CARE CENTER IN INDIA

The study was approved by the PGIMER Ethics committee. The medical records of 60 children <12 years of age attending Glaucoma clinic of Advanced Eye Centre, PGIMER Chandigarh from January 2006 to December 2012, who had secondary glaucoma as a result of ocular trauma and had minimum 6 months follow-up were reviewed, retrospectively. The parameters studied in this study included patient's demographical profile, various modes of injury, time of onset of glaucoma after injury, clinical presentation and management. The outcome in terms of intraocular pressure and visual acuity where possible, was recorded.

Out of the total 292 pediatric glaucoma cases enrolled in our clinic during this period, sixty six were post-traumatic. Four patients had insufficient information to be included under analysis and two were lost to follow-up before 6 months and hence were not included for analysis. Sixty patients were finally enrolled for the study. Hence, the calculated incidence of glaucoma due to trauma was found out to be 20%. Mean age of presentation of traumatic glaucoma was 10.8 ± 2.5 years. Males (85.5%) were affected more than females. The mean time after injury when the glaucoma was first diagnosed (elsewhere or at our center was 1.67 ± 1.7 weeks (24 hours to 6 weeks). After trauma 83.3% presented with glaucoma within 4 weeks. Fire cracker injury was the most common cause of trauma in children in our cohort, accounting for 16 cases (26.7%), followed by injury with stone in 12 (20.0%) children. A total of 35 patients (57.3%) presented with hyphema, subluxation of lens occurred in 3 (5%) patients. Angle recession was seen in 10 (16.6%) patients. The IOP at presentation was 33.7 ± 12.3 mm Hg which decreased to 12.90 ± 2.22 mm Hg at last follow-up. Out of 35 patients with hyphema, in 32 (91.4%), it resolved with conservative management. Three patients required drainage of the hyphema to control IOP. Seven patients had vitreous hemorrhage with hyphema. In 4 patients, it resolved spontaneously while 3 patients required surgery 6 patients developed advanced glaucomatous changes and required surgery Out of 6, two patients had presented late to our clinic (>6 months after injury) 2 patients had persistently high IOP >40 mm Hg for more than 8 weeks period. Outcome in terms of IOP control was good in 91.7% patients, satisfactory in 5.0% and poor in 3.3% patients**.** Overall, 54 (86.66%) patients could be managed conservatively while the rest required glaucoma filtration surgery. In this study, patients with very high IOP even upto 60 mm Hg did well when presented early and managed properly in time where as patients with moderately high pressure developed glaucomatous changes when IOP was not controlled well in time. This data concludes that traumatic glaucoma in children, if managed appropriately in time, has a good outcome.

## CONCLUSION

Trauma is an important cause of ocular morbidity. The clinical course of the patient after trauma may range from subtle signs to a series of sight threatening complications, which are difficult to manage. In the assessment after ocular trauma, it is important to identify and treat secondary conditions such as post-traumatic glaucoma that may adversely affect visual outcome to minimize any additional damage that may occur. Most eye injuries in children are preventable but also more serious. It is, hence, useful to have an insight into the mechanisms by which ocular trauma causes glaucoma, clinical profile of such cases and an approach to their management.

## References

[1] Brophy M, Sinclair SA, Hostetler SG, Xiang H (2006). Pediatric eye injury-related hospitalizations in the United States.. Pediatrics.

[2] Desai P, MacEwen CJ, Baines P, Minassian DC (1996). Incidence of cases of ocular trauma admitted to hospital and incidence of blinding outcom. Br J Ophthalmol.

[3] Abbott J, Shah P (2013). The epidemiology and etiology of pediatric ocular trauma.. Surv Ophthalmol.

[4] May DR, Kuhn FP, Morris RE, Witherspoon CD, Danis RP, Matthews GP, Mann L (2000). The epidemiology of serious eye injuries from the United States Eye Injury Registry.. Graefes Arch Clin Exp Ophthalmol.

[5] Osman EA, Al-Fawaz N, Al-Otaibi AG, Al-Mansouri SM, Mousa A, Al-Mezaine HS (2013). Glaucoma after open globe injury at a tertiary care university hospital in Central Saudi Arabia. Cumulative incidence and risk factors.. Saudi Med J.

[6] Girkin CA, Mc Gwin G Jr, Morris R, Kuhn F (2005). Glaucoma following penetrating ocular trauma: a cohort study of the United States Eye Injury Registry.. Am J Ophthalmol.

[7] Moren Cross J, Griffin R, Owsley C, McGwin G Jr (2008). Pediatric eye injuries related to consumer products in the United States, 1997-2006. J AAPOS..

[8] Abbott J, Shah P (2013). The epidemiology and etiology of pediatric ocular trauma.. Surv Ophthalmol.

[9] Strahlman E, Elman M, Daub E, Baker S (1990). Causes of pediatric eye injuries. A population-based study.. Arch Ophthalmol.

[10] Jaison SG, Silas SE, Daniel R, Chopra SK (1994). A review of childhood admission with perforating ocular injuries in a hospital in northwest India.. Indian J Ophthalmol.

[11] Agapitos PJ, Noel LP, Clarke WN (1987). Traumatic hyphema in children.. Ophthalmology.

[12] DeRespinis PA, Caputo AR, Fiore PM, Wagner RS (1989). A survey of severe eye injuries in children.. Am J Dis Child.

[13] Wright KW, Wright KW, Spiegel PH (2003). Pediatric ocular trauma: Pediatric hyphema.. Pediatric Ophthal mology and Strabismus..

[14] Spirn MJ, Lynn MJ, Hubbard GB 3rd (2006). Vitreous hemorrhage in children.. Ophthalmology.

[15] Sihota R, Kumar S, Gupta V, Dada T, Kashyap S, Insan R, Srinivasan G (2008). Early predictors of traumatic glaucoma after closed globe injury: trabecular pigmentation, widened angle recess, and higher baseline intraocular pressure.. Arch Ophthalmol.

